# Supportive care for talquetamab-related dysgeusia in multiple myeloma: mixed-methods evidence, nutrition-focused flowchart, and digital companion concept

**DOI:** 10.1007/s00520-026-10863-z

**Published:** 2026-06-17

**Authors:** Anna Fleischer, Magdalena Roll, Franziska Panther, Jessica Peter, Sofie Kadel, Christine Riedhammer, Patrick-Pascal Strunz, Christoph Schaefers, Anja Gesierich, Julia Mersi, Johannes Waldschmidt, Götz Gelbrich, K. Martin Kortüm, Hermann Einsele, Imad Maatouk, Leo Rasche

**Affiliations:** 1 Department of Internal Medicine II, University Hospital Würzburg, Oberdürrbacherstr. 6, 97080 Würzburg, Germany; 2 Department of Hematology, Oncology and Bone Marrow Transplantation With Section Pneumology, University Medical Center Hamburg-Eppendorf, Hamburg, Germany; 3 Department of Dermatology, Venereology and Allergology, University Hospital Wuerzburg, Josef-Schneider-Str. 2, 97080 Würzburg, Germany; 4 Institute of Biometry and Epidemiology, University Hospital of Würzburg, Würzburg, Germany

**Keywords:** Dysgeusia, GPRC5D, Multiple myeloma, Talquetamab, Mixed-methods, Nutrition, Taste profile, Patient-reported outcomes, Digital health, Supportive care

## Abstract

**Purpose:**

Talquetamab (TAL) frequently induces a distinctive dysgeusia that diminishes eating enjoyment, oral intake, and quality of life in patients with multiple myeloma (MM). Patient-centered, evidence-based nutritional guidance tailored to TAL-related sensory phenotypes is scarce. This mixed-methods study aimed to characterize patient experiences, identify preferred dietary adaptations, and translate these findings into a proposed exploratory clinical workflow and a digital companion support concept for future validation.

**Methods:**

A prospective, single-center exploratory mixed-methods study was conducted in talquetamab-treated patients with multiple myeloma who reported new-onset taste change (*n* = 25). Patient-reported dysgeusia, xerostomia symptoms, dietary experiences, and coping strategies were captured using a structured questionnaire with free-text fields. Taste change and xerostomia were assessed by patient report; objective psychophysical taste testing and sialometry were not performed in this cohort. Free-text responses were analyzed by two independent coders using reflexive thematic analysis with iterative consensus. Quantitative data were summarized descriptively. Integrated findings were translated in interprofessional focus groups into two exploratory supportive-care outputs: a proposed clinical workflow and a digital companion blueprint for future validation.

**Results:**

Patients described heterogeneous and individualized taste disturbances, including reduced or unpleasant perception of sweet flavors, bitter/sour aversions, spice-related mucosal sensitivity, and reduced enjoyment of meals. Patient-reported xerostomia symptoms aggravated intolerance to dry or fibrous foods and impaired swallowing comfort. Frequently reported coping strategies included mild herbs and aroma cues, umami-rich additions, sauce- or soup-based texture modification, temperature and plating adjustments, saliva-supportive measures, and environmental or behavioral strategies. Based on these findings, we developed an exploratory supportive-care workflow incorporating cycle-based symptom screening, prospective use of validated taste assessment where feasible, structured xerostomia and nutritional-risk assessment, phenotype-oriented dietary suggestions, safety escalation, and follow-up. A digital companion blueprint (GUSTABOR) was conceptualized to support future individualized dietary guidance after usability and effectiveness testing.

**Conclusions:**

Talquetamab-related dysgeusia is clinically meaningful, heterogeneous, and closely linked to oral dryness symptoms, food texture tolerance, eating enjoyment, and social participation. The proposed workflow and digital companion blueprint should be interpreted as exploratory supportive-care concepts derived from patient-reported experience, not as validated clinical tools. Prospective multicenter studies using validated taste instruments, objective salivary-flow assessment, nutritional endpoints, and implementation outcomes are required before routine clinical adoption.

**Implications for cancer survivors:**

Structured symptom screening, validated taste assessment where feasible, and individualized nutrition support may help identify patients at risk for reduced intake, weight loss, and impaired quality of life during talquetamab therapy. Digital support could broaden access to tailored guidance, but should be implemented only after prospective evaluation of usability, safety, resource requirements, and clinical benefit.

## Introduction

MM treatment is evolving rapidly with the introduction of T-cell-redirecting bispecific antibodies such as TAL, a first-in-class GPRC5D × CD3 bispecific antibody that has demonstrated robust and durable responses in heavily pretreated relapsed/refractory MM (RRMM) [1–3]. Beyond its antimyeloma activity, TAL is characterized by a distinct pattern of GPRC5D-related oral adverse events, most notably dysgeusia and xerostomia, which occur in a substantial proportion of treated patients [4–7].

The mechanistic basis for TAL-related dysgeusia is thought to be linked to the restricted yet high expression of GPRC5D on malignant plasma cells and on selected normal keratinizing epidermis tissues, including hair follicles, nail matrix, eccrine glands and the filiform papillae of the tongue, while showing minimal expression in most other normal tissues [3, 6]. This expression pattern supports the prevailing concept that TAL-induced oral toxicities represent *on-target*, *off-tumor* effects rather than nonspecific off-target damage [3, 5, 6]. However, the precise biological pathways by which GPRC5D engagement in filiform papillae—structures that do not contain taste buds—translate into the complex qualitative taste disturbances reported by patients remain only partially understood, and currently available experimental and clinical data are insufficient to fully explain the spectrum and dynamics of TAL-related dysgeusia and xerostomia [3, 4].

Recent prospective and observational studies indicate that these sensory disturbances can profoundly affect eating enjoyment, oral intake, body weight, and multiple dimensions of health-related quality of life [8, 9]. However, structured, patient-centered, and evidence-based strategies to prevent and manage TAL-related taste and salivary disturbances remain limited. Existing recommendations largely rely on expert consensus and generic supportive-care measures rather than systematically collected patient experience data [5, 6, 10].

Assessment of cancer- and treatment-related taste disturbance is methodologically challenging because subjective dysgeusia, objective gustatory dysfunction, olfactory dysfunction, oral pain, xerostomia, hyposalivation, nausea, mucositis, and nutritional decline may overlap. Several validated or structured approaches are available and should inform future studies, including psychophysical Taste Strips for sweet, sour, salty, and bitter identification [11, 12], patient-reported instruments such as the Chemotherapy-induced Taste Alteration Scale (CiTAS) [13], PRO-CTCAE taste-change and dry-mouth items [14], the EORTC QLQ-OH15 oral-health module [15], and objective salivary-flow assessment for suspected hyposalivation [16, 17]. The present study was therefore designed as an exploratory patient experience study and not as a validation study of a diagnostic taste instrument.

As TAL moves from late-line monotherapy into broader use in combination regimens and earlier treatment lines within the relapsed/refractory setting—including phase 2 and 3 trials such as MonumenTAL-3 and several combination programs (e.g. daratumumab- and teclistamab-based regimens)—the clinical relevance of its oral toxicities continues to increase [5, 6, 18]. Dysgeusia and xerostomia are not only common but also compromise patients’ ability to maintain adequate oral intake and to participate in social eating situations, thereby amplifying the overall burden of disease and treatment [4, 8, 9].

In the MonumenTAL-1 trial, oral toxicities were among the most frequent GPRC5D-associated adverse events. They were, however, predominantly low grade (Common Terminology Criteria for Adverse Events grade 1–2) and led to dose modifications or treatment discontinuation in only a minority of cases [1, 2, 6]. This characteristic pattern of chronic, predominantly low grade but functionally relevant symptoms underscores the need for structured, proactive, and personalized supportive care that goes beyond generic dietary advice. Current practice guidance and nursing toolkits largely recommend broad measures such as intensified seasoning, temperature and texture variation, saliva substitutes, sugar-free lozenges, and optimized oral hygiene. Yet, they pay limited attention to the considerable interindividual heterogeneity in sensory profiles, texture aversions, comorbidities, and cultural eating practices that shape patients’ real-world adaptation strategies [5, 6, 10].

This mixed-methods study aims to address this critical gap by systematically characterizing the lived experiences of patients with TAL-induced dysgeusia, delineating their preferred dietary, sensory, and behavioral adaptations, and translating these insights into actionable tools for clinical practice.

## Methods

### Design and setting

This prospective, single-center exploratory mixed-methods study was conducted over 12 months at the myeloma center of the University Hospital of Wuerzburg, Germany. The study aimed to characterize patient-reported talquetamab-related taste disturbance and to generate hypotheses for supportive-care management. The study was not designed or powered to validate a clinical tool or to test intervention efficacy. The clinical context reflected subcutaneous talquetamab regimens reported in MonumenTAL-1 (0.4 mg/kg weekly; 0.8 mg/kg every 2 weeks) to support clinical interpretability [6]. The protocol prespecified quantitative description, qualitative analysis of patient narratives, and an a priori integration phase in interprofessional focus groups.

### Participants

Eligible participants were adults with multiple myeloma receiving TAL who reported new-onset dysgeusia after therapy initiation. Exclusion criteria were lack of informed consent or TAL exposure shorter than 3 months. Consecutive eligible patients were approached during routine care. The final sample comprised 25 participants. For the qualitative component, sampling adequacy followed the information power principle [19]. Comorbidities and concomitant factors with potential relevance to taste, oral dryness, appetite, swallowing, or nutritional risk were extracted descriptively from routine clinical records and patient reports where available. These included diabetes, renal dysfunction, gastrointestinal symptoms, oral or dental problems, mucositis, prior antimyeloma therapies, concomitant medications with xerogenic potential, and baseline nutritional concerns. Because of the modest sample size and exploratory aim, these variables were not used for adjusted inferential modeling; they were considered contextual factors when interpreting patient-reported symptoms.

### Data collection

A structured, self-designed questionnaire was used to capture demographics, patient-reported taste-related experiences, xerostomia symptoms, food preferences and aversions, coping strategies, dining environment, and social context. Taste change was determined by patient self-report after initiation of talquetamab and was explored using structured questions and free-text prompts. Xerostomia was assessed as the subjective sensation of dry mouth; objective hyposalivation or salivary-gland hypofunction was not determined because unstimulated or stimulated whole-saliva flow rates were not measured in this cohort [16, 17]. A brief cognitive pretest with three patients ensured clarity and resulted in minor wording refinements. Free-text responses addressing dietary experiences and coping strategies were analyzed qualitatively and subsequently integrated with descriptive quantitative findings.

### Definitions and measurement boundaries

In the revised manuscript, dysgeusia refers to patient-reported abnormal or altered taste perception during talquetamab therapy. Xerostomia refers to the subjective symptom of oral dryness. Hyposalivation or salivary-gland hypofunction refers to objectively reduced salivary output and was not measured in the present study [16, 17]. The proposed clinical workflow therefore recommends future structured assessment using validated taste tests [11, 12], patient-reported oral-symptom instruments [13–15], and objective salivary-flow measurement where clinically feasible [16, 17], but these measures should not be interpreted as having been applied in the current dataset.

### Outcomes

The primary descriptive outcome was the qualitative characterization of patient-reported talquetamab-associated taste disturbance and patient-preferred coping strategies. This outcome was selected because the clinical gap concerns patient-centered supportive-care needs and real-world dietary adaptation rather than drug efficacy. Secondary exploratory outcomes included contextual modifiers of eating (environmental and social factors), xerostomia-related modifiers of food texture tolerance and swallowing comfort, signals of nutritional risk, and the feasibility of deriving two non-validated conceptual outputs: a proposed clinical supportive-care workflow and a digital companion blueprint. No inferential efficacy endpoint was prespecified.

### Quantitative analysis

We summarized variables using descriptive statistics (means with standard deviations, medians with interquartile ranges, and counts with percentages). No inferential hypothesis testing was prespecified given the implementation-oriented translational aim. Missing data were reported case-wise without imputation. In all analyses, data were processed using IBM SPSS Statistics, Version 29.0 (IBM Corp., Armonk, NY, USA).

### Qualitative analysis

We conducted reflexive thematic analysis in six phases: familiarization, initial coding, theme generation, theme review, theme definition, and reporting. Two coders analyzed free-text responses independently and resolved differences through iterative consensus discussions. The coders were not externally calibrated against a formal qualitative coding standard; this is acknowledged as a limitation. Reflexivity memos and audit notes were maintained to document analytic decisions and to surface potential influences related to clinical roles and prior expectations. Sampling adequacy was interpreted using the information power principle, taking into account the focused research question, specificity of the sample, and pragmatic supportive-care aim [20, 21].

### Mixed-methods integration and translation

Quantitative descriptive findings and qualitative themes were integrated in interprofessional focus groups involving hematology/oncology, dietetics, psycho-oncology, and nursing. The purpose of integration was not to validate an intervention, but to translate patient-reported needs into a reproducible supportive-care proposal for future evaluation. The focus groups mapped patient-reported symptom patterns and coping strategies onto clinically actionable domains: screening, taste assessment, xerostomia/salivary assessment, nutritional-risk triage, red-flag escalation, phenotype-oriented dietary suggestions, follow-up, and digital-support requirements.

### Ethics

The institutional review board of the University Hospital Würzburg approved the study (AZ 119/22), and procedures complied with the Declaration of Helsinki. Clinical trial number: not applicable. All participants provided written informed consent.

### Data management and availability

Data were pseudonymized with a separately stored key and held on secure servers with access controls. Data and analysis templates are available from the corresponding author upon reasonable request in line with consent and data-protection requirements.

## Results

### Participant characteristics

The cohort comprised 25 patients with multiple myeloma (17 male, 8 female; mean age 63.9 years, range 48–85) receiving talquetamab who reported taste change after therapy initiation. Advanced disease was frequent (44.0% ISS stage 3). Comorbidities, prior therapies, concomitant medications, oral symptoms, and nutritional concerns were heterogeneous, reflecting a real-world relapsed/refractory myeloma population. Because the study was exploratory and not powered for subgroup inference, these factors were considered descriptively rather than modeled as covariates. Minimal baseline characteristics are summarized in Table 1.

**Table 1:** Participant characteristics (minimal baseline set; TAL cohort, *n *= 25)

Variable	Value
Age, years	Mean 63.9; range 48–85
Sex	Male 17 (68.0%); female 8 (32.0%)

Data shown are the minimal baseline variables available for this exploratory analysis. Comorbidities, concomitant medications, prior therapies, and oral/nutritional factors were heterogeneous and considered descriptively where available, but the study was not powered for adjusted subgroup analyses. Comprehensive cohort characteristics are reported in the companion paper: Fleischer A, et al. *Talquetamab-Related Dysgeusia in Multiple Myeloma Compared to BCMA-Targeted Bispecifics and High-Dose Melphalan.* Cancer Medicine. 2025;14(23):e71401. https://doi.org/10.1002/cam4.71401. PMID: 41360668; PMCID: PMC12685487

### Reported dietary challenges

**Fig. 1:**
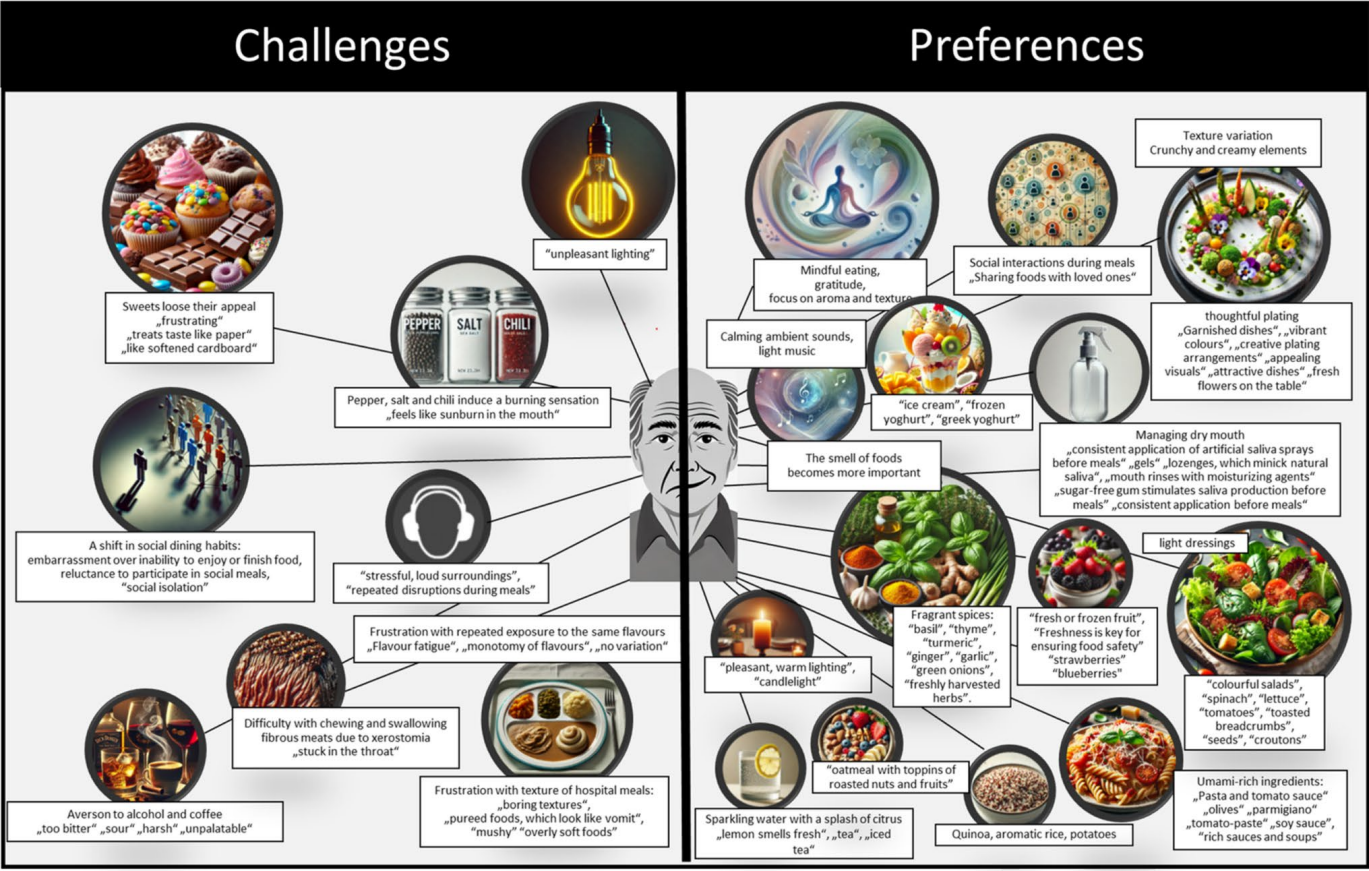
Dietary challenges and preferences of TAL-treated MM patients. **A** Summarizes patient-reported challenges (sweet aversion, spice sensitivity, xerostomia-linked texture intolerance, hospital meal texture issues, environmental stressors, flavor fatigue, reduced social dining). **B** Summarizes preferred strategies (umami focus, mild herbs/aroma cues, texture contrast, xerostomia aids, temperature/presentation cues, mindful eating, fresh fruits/crisp salads, sparkling water/citrus, sugar-free gum)

Patients reported pronounced and highly individualized alterations in taste perception, with frequent aversion to sweet flavors, which were described as bland, unpleasant, or nausea-inducing. Bitter and sour aversions, mucosal sensitivity to spices, and reduced meal satisfaction were also common. Hospital meals were often criticized for poor texture and limited multisensory appeal, with puréed foods being particularly aversive for some patients. Patient-reported xerostomia symptoms aggravated difficulties with dry or fibrous foods, such as meat, by impairing bolus formation and swallowing comfort. Many patients compensated by taking frequent sips, adding sauce, choosing soups, or preferring moist and softer textures. Environmental stressors, including harsh lighting, elevated noise, and frequent interruptions, further reduced appetite and enjoyment. Figure 1 summarizes the main patient-reported challenges and preferred adaptations.

### Dietary preferences and positive adaptations

Patients identified multisensory strategies as helpful, including mild fragrant herbs (e.g., basil, thyme), umami-rich additions (e.g., tomato, mushrooms, soy, parmesan), and adjustments to texture, temperature, and plating. Many patients reported that their sense of taste was significantly impaired, while their sense of smell remained largely intact. As a result, they emphasized the importance of aromas perceived through the nose in enhancing the overall eating experience. Warm, indirect lighting and calming ambient music were perceived as supportive. Non-overstimulating social interactions facilitated a sense of normalcy during meals. Mindful eating practices redirected attention to aroma, temperature, and mouthfeel. To mitigate xerostomia and stimulate salivation, patients frequently used sparkling water with citrus, herbal teas, and sugar-free chewing gum.

To reduce difficulty swallowing, many patients emphasized the benefit of adding a large amount of sauce to every dish and stated that they regularly consumed soups. Preferred foods included fresh fruits (notably berries) and vibrant, crisp salads. Umami-forward dishes (e.g., pasta with tomato sauce, olives, miso/soy) sometimes remained palatable. These reported preferences are also visualized in Fig. 1.

### Patient inventory of coping strategies

Table 2 presents a structured inventory of patient-derived interventions along the preparation and consumption continuum, including fresh ingredient selection, marination, texture variation (crunchy/creamy contrasts), umami focus, balanced seasoning across modalities, mild herbs/aromatics, olfactory stimulation (e.g., citrus zest, gentle sautéing), mindful eating, presentation/plating, dining-environment optimization, high-calorie shakes/supplements, and rotation to counter flavor fatigue. Xerostomia-directed tactics (e.g., saltwater rinses, artificial saliva) and safeguards to maintain adequate caloric intake are integrated.

**Table 2:** Patient inventory: strategies to mitigate taste disturbances in patients treated with TAL

Strategy	Short description/rationale/narrative summary
Use of fresh ingredients	The preference for high-quality, fresh ingredients was consistently emphasized by patients experiencing taste disturbances during TAL therapy. Fresh ingredients like crisp vegetables, ripe fruits, and freshly harvested herbs were favored not only for their vibrant flavors but also for their natural aromas and textures. Fresh produce and herbs helped mitigate unpleasant taste disturbances by providing flavors that were more discernible and satisfying. For example, patients preferred fresh strawberries or blueberries as snack options or breakfast toppings over processed alternatives. The use of fresh garlic, ginger, and green onions contributed significantly to flavor depth, particularly in savory dishes. Moreover, freshness was key for ensuring food safety in patients with reduced taste perception, with patients favoring newly sourced ingredients over preserved counterparts to avoid off-putting smells and tastes
Marination	Marinating proteins and vegetables prior to cooking added layers of flavor that compensated for taste disturbances
Routine use of salivary Substitutes and mouth rinses	Managing dry mouth (xerostomia) is critical for patients undergoing TAL therapy Routine use of interventions like saltwater rinses, artificial saliva, and sugar-free gums/candy/lozenges or dry mouth gel before bedtime helped alleviate dryness and improved taste sensation. Saltwater rinses were commonly used before meals to cleanse the mouth and stimulate saliva production, while specialized mouth rinses with moisturizing agents provided extended relief. Artificial saliva sprays, gels, and lozenges mimicked natural saliva, easing chewing and swallowing. Consistent application of these strategies, particularly before meals, resulted in a more pleasant eating experience In case of dysphagia, dietary modifications such as small bites, soups, and vegetable or non-sweet smoothies were used to facilitate safer and more comfortable swallowing
Texture variation	Incorporating a variety of textures helped patients maintain a satisfying eating experience. The contrast between crunchy and creamy elements added sensory richness to meals, compensating for altered taste perceptions. Patients often added toppings like toasted breadcrumbs, roasted nuts, or seeds to dishes for a satisfying crunch. Creamy components, such as mashed potatoes, sauces, or yogurt, were also essential for improving the palatability of meals. This focus on texture was especially effective when flavors were muted or altered, as it allowed patients to overlook taste changes while still finding enjoyment in their food
Umami-rich ingredients	Umami-rich ingredients, such as tomato paste, mushrooms, soy sauce, and Parmesan cheese, were particularly effective in enhancing flavor perception for patients with reduced taste sensitivity. The savory depth of umami provided a satisfying fullness that was easier to detect when other taste perceptions were muted. Incorporating these ingredients into dishes like soups, stews, and pasta enhanced the overall richness and complexity of meals, making them more enjoyable despite taste disturbances
Balanced seasoning	Balancing the five main taste elements—sweet, salty, sour, bitter, and umami—was key to enhancing the appeal of meals for patients with varying taste sensitivities. Ingredients like honey, soy sauce, tomato paste, lemon juice, and Parmesan cheese were used to create a rich flavor profile. Patients also experimented with contrasting flavors (e.g., sweet and salty, acidic and creamy) to stimulate the palate and make meals more interesting. Pairing tart fruits with rich, savory foods was another common strategy for enhancing flavor
Spices and aromatics	Experimenting with a variety of spices and mild herbs added much-needed complexity and interest to meals. Mild herbs like basil and thyme, along with aromatics like garlic and ginger, provided depth and warmth to dishes. Smoked paprika and turmeric were particularly appreciated for introducing unique flavor notes. Patients found that even small additions of spices and aromatics could make a significant difference in overall meal enjoyment. However, as too much spice caused pain in some patients, spices should always be used with caution and dosed according to individual preference
Olfactory stimulation	Enhancing the aroma of dishes played a crucial role in improving the eating experience for patients with altered taste. Citrus zest (e.g., lemon, lime, orange) and fresh herbs were commonly used to add a refreshing and vibrant scent to meals. Sautéing garlic, ginger, and onions at the start of cooking also released potent aromas that created a sense of anticipation, making meals more enjoyable even before the first bite. Patients reported that paying attention to the aroma of food helped compensate for diminished taste sensations
Mindful eating and savoring	Mindful eating practices helped amplify sensory appreciation of food by focusing on subtle qualities like aroma and texture. Some TAL patients reported that they were encouraged by nutritionists to take small bites, chew slowly, and fully engage with the sensory details of each meal. This approach not only enhanced enjoyment but also reduced anxiety around eating. Techniques like taking deep breaths before meals, expressing gratitude, and avoiding distractions allowed patients to reconnect with their food in a more satisfying way
Presentation and plating	The visual appeal of a dish became increasingly important as taste diminished. Visually attractive meals, characterized by vibrant colors and thoughtful plating, stimulated appetite and created a sense of anticipation. Patients favored colorful salads, garnished dishes, and creative plating arrangements. The use of bright dinnerware and contrasting sauces contributed to a more positive relationship with food, helping patients look forward to meals despite taste changes
Optimizing the dining environment	Creating a pleasant dining atmosphere was key to enhancing meal experiences. Patients described how adjustments like soft lighting, calming music, and decorative table settings made meals more inviting. Social interactions during meals, such as sharing food with loved ones, were particularly valued for fostering a sense of connection and offsetting the frustrations of altered taste. For those dining alone, setting a table with fresh flowers or arranging plates with extra care contributed to a more enjoyable experience
Incorporating high-caloric shakes and nutritional supplements	High-calorie shakes and nutritional supplements were essential for patients with decreased appetite or difficulty consuming regular meals. Ready-made nutritional drinks and homemade smoothies provided concentrated nutrition in a palatable format. Patients often preferred thicker, creamier textures, which were more satisfying than thin liquids. Blending ingredients like yogurt, nut butter, and protein powder allowed patients to tailor shakes to their taste preferences, ensuring they received adequate nutrition
Adapting to flavor fatigue	Patients noted that flavors once enjoyed became unpalatable after repeated exposure. To alleviate flavor fatigue, patients rotated through different meals and introduced new foods to keep their diet interesting. Experimenting with previously unfamiliar ingredients or recipes helped rekindle interest in eating and reduced the monotony associated with repeated flavors

### Focus groups and intervention development

**Fig. 2:**
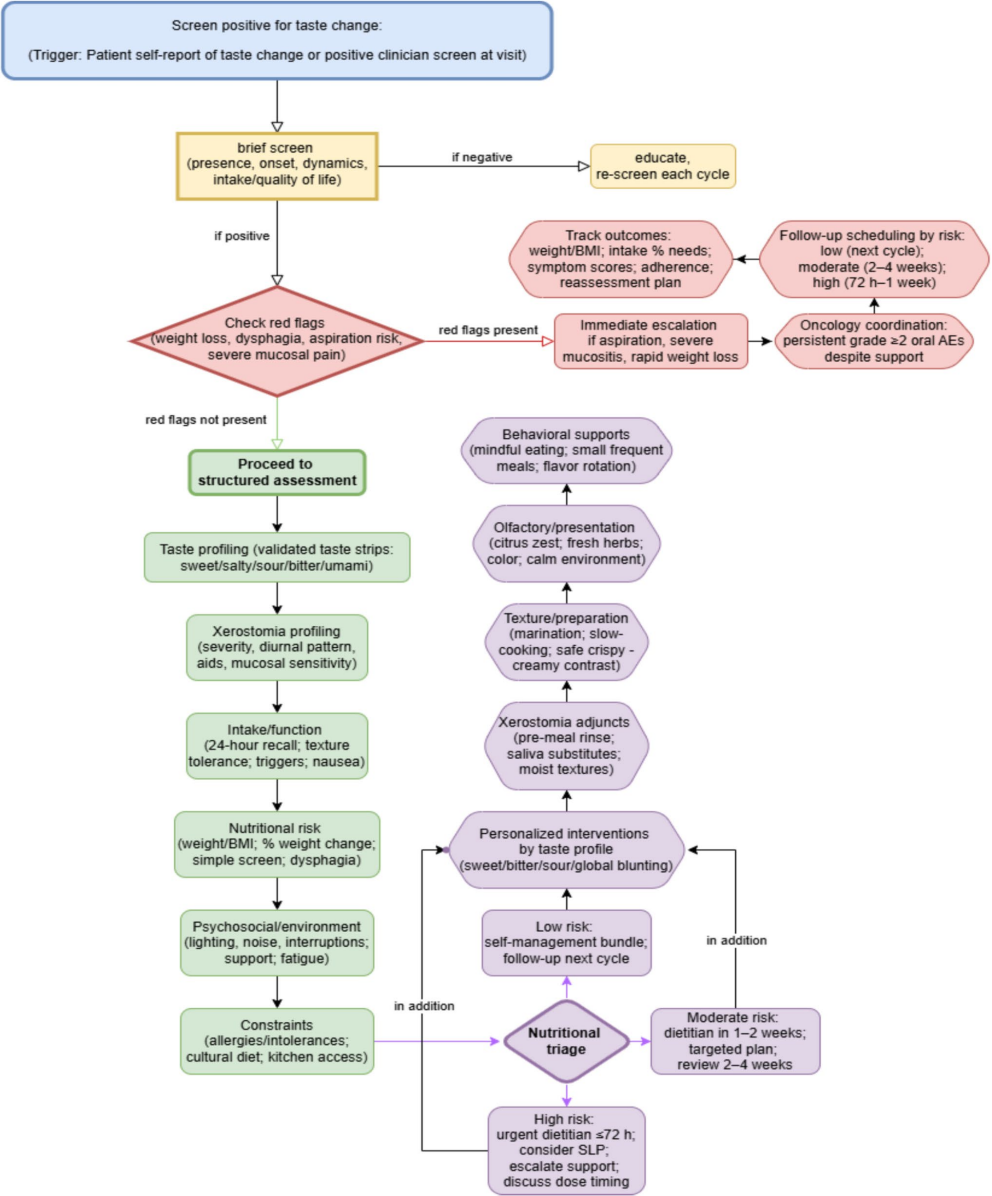
Proposed exploratory workflow for standardized assessment and supportive-care triage of talquetamab-related taste disturbance. The workflow incorporates cycle-based patient-reported symptom screening, prospective use of validated taste assessment where feasible, structured evaluation of xerostomia symptoms and nutritional risk, red-flag escalation, phenotype-oriented supportive-care suggestions, dietitian referral according to risk tier, and scheduled reassessment. The workflow is a proposal for future validation and should not be interpreted as a validated clinical tool

Interprofessional focus groups synthesized quantitative descriptions and qualitative themes into two exploratory outputs intended for future validation. First, a proposed clinical supportive-care workflow was developed to operationalize cycle-based symptom screening, prospective use of validated taste assessment where feasible [11, 12], structured assessment of xerostomia symptoms and, in future studies, objective salivary-flow testing [16, 17], nutritional-risk triage, red-flag escalation, and scheduled reassessment (Fig. 2). The workflow is a pragmatic proposal and should not be interpreted as a validated clinical instrument.

Second, we developed the concept for GUSTABOR, a digital companion blueprint intended to support nutritional counseling for patients with therapy-related taste disturbance. Conceptually, GUSTABOR would integrate patient-reported taste changes, validated taste profiles when available, oral dryness symptoms, BMI and weight trajectory, allergies, intolerances, kitchen resources, dietary patterns, and patient preferences to generate tailored dietary suggestions. The present manuscript describes the concept and clinical logic only. Usability, data protection, interoperability, safety, resource requirements, and clinical effectiveness are being evaluated in subsequent research and development work before any claim of routine implementation can be made.

To maintain reproducibility and avoid excessive resource burden, the focus groups favored a stepwise model: brief screening for all patients, structured supportive-care advice for low-risk patients, dietitian referral for moderate-risk patients, and urgent multidisciplinary escalation for red flags such as clinically relevant weight loss, dysphagia, aspiration risk, mucositis, dehydration, or severely reduced oral intake.

## Discussion

TAL-associated taste disturbances constitute a clinically meaningful, multidomain symptom complex with measurable effects on nutritional intake, social participation, and quality of life, in line with safety and activity data from MonumenTAL-1, where oral adverse events—most commonly dysgeusia and xerostomia—were frequent and predominantly low grade [1, 2, 4]. The symptom configuration observed in this study—predominant aversion to sweet, frequent bitter and sour aversions, spice-related mucosal sensitivity, and xerostomia-linked intolerance to dry or fibrous textures—maps onto the documented oral-toxicity domain under TAL and underscores the need for structured, phenotype-sensitive supportive care that is integrated into routine practice [4, 7, 8].

Supportive-care recommendations for taste disturbance require well-defined and reproducible assessment criteria. Our findings should therefore be interpreted in light of the available measurement landscape. Psychophysical Taste Strips provide a validated bedside approach for quantifying identification of sweet, sour, salty, and bitter qualities [11, 12]. Patient-reported tools, including CiTAS [13], PRO-CTCAE taste-change and dry-mouth items [14], and the EORTC QLQ-OH15 oral-health module [15], can capture symptom burden and functional impact. For oral dryness, xerostomia should be distinguished from objectively measured hyposalivation, which requires salivary-flow assessment [16, 17]. The present study did not include these objective measures; accordingly, our flowchart recommends them for future validation rather than presenting them as completed measurements in the current cohort.

To contextualize the specificity of TAL-related taste disturbances within the broader class of T-cell-redirecting bispecifics, it is noteworthy that dysgeusia has also been observed with tarlatamab, a DLL3 × CD3 bispecific T-cell engager approved for adults with extensive-stage small cell lung cancer after progression on or after platinum-based chemotherapy. In the phase 2 DeLLphi-301 trial, any-grade dysgeusia occurred in approximately 29% of tarlatamab-treated patients and featured among the most common treatment-emergent adverse events [22]. This convergence suggests that gustatory disturbance may represent a cross-agent signal relevant to supportive-care pathways beyond GPRC5D-targeting therapy and supports target-agnostic, phenotype-based management strategies for T-cell engagers in general.

A central contribution of this study is the structured translation of patient-reported experience into an exploratory supportive-care workflow. The workflow is intentionally pragmatic: it begins with brief cycle-based symptom screening and then escalates to structured assessment of taste, xerostomia symptoms, oral intake, nutritional risk, safety red flags, and follow-up needs. This approach may help clinicians move beyond generic dietary advice, but the workflow should not yet be considered validated or proven effective. Its feasibility, reliability, inter-rater reproducibility, patient acceptability, effect on nutritional intake, and impact on quality of life require prospective evaluation.

First, the standardized clinical flowchart operationalizes cycle-based screening, validated taste-strip profiling across modalities, structured assessment of xerostomia, oral intake and nutritional risk, phenotype-matched interventions, explicit safety-escalation rules, and scheduled reassessment. This proposed workflow is intended to address limitations of generic guidance by aligning recommendations with the individual sensory phenotype and xerostomia-constrained texture tolerance, while deliberately incorporating environmental and behavioral modifiers that patients identified as impactful. Second, the digital blueprint specifies how validated taste profiles, nutritional requirements, and patient-reported outcomes can be synthesized into tailored, adaptive suggestions that are compatible with clinical workflows and scalable for decentralized care. These translational elements are congruent with the adverse-event profile and pragmatic supportive measures reported in clinical development and post-approval management of TAL, including mouth rinses, salivary substitutes, dose modifications, and changes in dosing frequency where clinically indicated [5, 6, 10].

Implementation feasibility is a major consideration. Personalized supportive care can become time-consuming and resource-intensive if it requires lengthy assessments, repeated dietitian input, specialized taste testing, or complex digital infrastructure for every patient. A scalable model should therefore be tiered: brief screening for all talquetamab-treated patients, targeted use of validated taste and salivary assessment when symptoms are clinically relevant [11–13, 15–17], automated or semi-automated low-risk advice, and dietitian or multidisciplinary escalation only for patients with weight loss, reduced intake, dysphagia, dehydration risk, mucositis, severe distress, or persistent functional impairment [16, 23]. Digital tools may reduce burden by standardizing intake and generating preliminary suggestions, but they also introduce requirements for usability testing, clinician oversight, interoperability, data protection, maintenance, reimbursement, and equitable access for patients with limited digital literacy.

Mechanistic statements are intentionally cautious. Expression of GPRC5D in keratinized oral structures, including filiform papillae of the tongue, supports a biologically plausible link to oral sensory perturbations [6, 24]. However, causal connections to modality-specific taste-receptor disruption remain insufficiently resolved and warrant targeted investigation [24]. The high prevalence and severity of dysgeusia in TAL-treated patients, including real-world cohorts where taste changes are a leading reason for treatment consideration or discontinuation [7], underscores the importance of elucidating these mechanisms. Although GPRC5D has been primarily identified as an antigenic target on malignant plasma cells, immunohistochemical data indicate GPRC5D immunoreactivity in non-malignant, cornified or papillary structures within the oral cavity and tongue [25]. This raises the hypothesis that an on-target, off-tumor effect of TAL on GPRC5D-expressing healthy oral structures could contribute to the sensory disturbance. Nevertheless, this remains speculative, as histological analyses of GPRC5D expression within classical taste buds are currently lacking, and no direct link between GPRC5D modulation and specific taste-receptor pathways has yet been demonstrated [6].

The approach presented here aligns with broader trends in supportive oncology toward routine PROs, hybrid (in-person and digital) care models, and explicit attention to patient experience within value-based care frameworks. Randomized trials and large implementation studies have shown that systematic PRO-based symptom monitoring during cancer treatment can improve quality of life, reduce emergency visits and hospitalizations, and in some settings even prolong overall survival [26, 27]. Embedding structured triggers into electronic health records—such as weight loss, insufficient caloric intake, or deterioration in taste-related PRO items—could enable earlier identification of patients at high risk for malnutrition and help standardize timely referrals to nutritional and psycho-oncological support. The proposed flowchart and digital blueprint are compatible with routine integration into electronic health records and ePRO platforms, allowing automated triggers based on weight loss, reduced intake, or worsening taste-related PRO scores.

Transferability of these findings appears plausible for other patient populations experiencing a high prevalence of dysgeusia, such as those undergoing head-and-neck radiotherapy, tyrosine kinase inhibitor therapy, or immunotherapies that affect oral tissues [28–30]. Patients treated with high-dose melphalan and autologous stem-cell transplantation or with proteasome-inhibitor-based regimens, who also experience clinically relevant taste alterations, may similarly benefit from structured, taste-profile-based interventions, given the shared downstream impact of dysgeusia on nutritional status and quality of life [7, 30]. Real-world experience with TAL further motivates proactive nutritional monitoring and supportive measures, including dose or frequency adjustments when clinically appropriate, to mitigate persistent oral adverse events and weight loss [31, 32].

This study has several limitations. First, the single-center design and modest sample size constrain generalizability and preclude adjusted analyses of comorbidities, concomitant medications, prior therapies, and other potential confounders. Second, taste change and xerostomia were assessed by patient report using a self-designed questionnaire; validated psychophysical taste testing [11, 12], CiTAS [13], PRO-CTCAE [14], EORTC QLQ-OH15 [15], and objective salivary-flow assessment [16, 17] were not applied in the current cohort. Third, the qualitative analysis was based on free-text responses and conducted by two coders who used iterative consensus but were not externally calibrated against a formal qualitative coding standard. Fourth, the proposed workflow and GUSTABOR blueprint are translational concepts derived from patient-reported experience and interprofessional synthesis; they are not validated clinical tools and cannot establish effectiveness. Fifth, cultural, socioeconomic, dental, olfactory, gastrointestinal, and nutritional determinants of taste preferences and eating behavior were not systematically assessed. Future multicenter studies should combine validated taste instruments, objective salivary assessment, structured nutritional endpoints, implementation metrics, and patient-centered outcomes to evaluate feasibility, reproducibility, cost, and clinical benefit.

Strengths include the focused investigation of an under-addressed supportive-care problem in talquetamab-treated patients, the integration of patient narratives with interprofessional clinical interpretation, and the generation of a practical framework that can be tested prospectively. The study surfaces multisensory adaptations spanning flavor, aroma, texture, temperature, plating, salivary support, and dining environment, which are often missed by routine toxicity assessments. Its main value is hypothesis generation and structured tool development, not validation of clinical effectiveness.

## Conclusion

Talquetamab-related dysgeusia is a clinically meaningful and heterogeneous symptom complex that may impair eating enjoyment, oral intake, social participation, and quality of life in patients with multiple myeloma. In this exploratory mixed-methods study, patient-reported experiences informed a proposed supportive-care workflow and a digital companion blueprint. These outputs should be viewed as structured concepts for future evaluation rather than validated clinical tools. Prospective multicenter studies using validated taste measures, objective salivary-flow assessment, nutritional outcomes, patient-reported symptom instruments, and implementation endpoints are needed before routine clinical adoption can be recommended.

## Data Availability

The data supporting the findings of this study are available from the corresponding author upon reasonable request.

## References

[1] Chari A et al (2022) Talquetamab, a T-cell-redirecting GPRC5D bispecific antibody for multiple myeloma. N Engl J Med 387(24):2232–2244 36507686 10.1056/NEJMoa2204591

[2] Chari A et al (2025) Safety and activity of talquetamab in patients with relapsed or refractory multiple myeloma (MonumenTAL-1): a multicentre, open-label, phase 1-2 study. Lancet Haematol 12(4):e269–e281 40090350 10.1016/S2352-3026(24)00385-5

[3] Shaver J, Horton D, Halford Z (2025) Targeting GPRC5D with talquetamab: a new frontier in bispecific antibody therapy for relapsed/refractory multiple myeloma. Ann Pharmacother 59(4):350–363 39192558 10.1177/10600280241271192

[4] Laheij A, van de Donk N (2023) Characterization of dysgeusia and xerostomia in patients with multiple myeloma treated with the T-cell redirecting GPRC5D bispecific antibody talquetamab. Support Care Cancer 32(1):20 38092979 10.1007/s00520-023-08233-0

[5] Chari A et al (2024) Clinical management of patients with relapsed/refractory multiple myeloma treated with talquetamab. Clin Lymphoma Myeloma Leuk 24(10):665-693.e14 38871558 10.1016/j.clml.2024.05.003

[6] Sandahl T, Sandahl PD et al (2025) Management of patients with relapsed/refractory multiple myeloma treated with talquetamab: highlights from pharmacists’ perspectives. J Adv Pract Oncol. 10.6004/jadpro.2025.16.7.15 40599198 10.6004/jadpro.2025.16.7.15 PMC12207530

[7] Fleischer A et al (2025) Talquetamab-related dysgeusia in multiple myeloma compared to BCMA-targeted bispecifics and high-dose melphalan. Cancer Med 14(23):e71401 41360668 10.1002/cam4.71401 PMC12685487

[8] Naqvi S et al (2024) Weight loss and dysgeusia in relapsed/refractory multiple myeloma patients treated with talquetamab. EJHaem 5(4):789–792 39157593 10.1002/jha2.971 PMC11327703

[9] Schinke C et al (2025) Talquetamab improves patient-reported symptoms and health-related quality of life in relapsed or refractory multiple myeloma: results from the phase 1/2 MonumenTAL-1 study. Cancer 131(14):e35927 40631904 10.1002/cncr.35927 PMC12239699

[10] Catamero D et al (2024) Nursing considerations for the clinical management of adverse events associated with talquetamab in patients with relapsed or refractory multiple myeloma. Semin Oncol Nurs 40(5):151712 39155155 10.1016/j.soncn.2024.151712

[11] Mueller C et al (2003) Quantitative assessment of gustatory function in a clinical context using impregnated “taste strips.” Rhinology 41(1):2–6 12677732

[12] Landis BN et al (2009) “Taste Strips” - a rapid, lateralized, gustatory bedside identification test based on impregnated filter papers. J Neurol 256(2):242–248 19221845 10.1007/s00415-009-0088-y

[13] Kano T (2013) Development and validation of a chemotherapy-induced taste alteration scale. Oncol Nurs Forum 40(2):E79–E85 23448748 10.1188/13.ONF.E79-E85

[14] Hagelstein V et al (2016) Validation of the German patient-reported outcomes version of the common terminology criteria for adverse events (PRO-CTCAE™). Ann Oncol 27(12):2294–2299 27681863 10.1093/annonc/mdw422 PMC6267864

[15] Hjermstad MJ et al (2016) International field testing of the psychometric properties of an EORTC quality of life module for oral health: the EORTC QLQ-OH15. Support Care Cancer 24(9):3915–3924 27113466 10.1007/s00520-016-3216-0

[16] Mercadante V et al (2021) Salivary gland hypofunction and/or xerostomia induced by nonsurgical cancer therapies: ISOO/MASCC/ASCO guideline. J Clin Oncol 39(25):2825–2843 34283635 10.1200/JCO.21.01208

[17] Hong C et al (2024) MASCC/ISOO Clinical Practice Statement: clinical assessment of salivary gland hypofunction and xerostomia in cancer patients. Support Care Cancer 32(8):551 39048727 10.1007/s00520-024-08691-0

[18] Miller KC, Hamadeh I, Tan CR (2025) Perspectives on talquetamab and its utility in the treatment of multiple myeloma: safety, efficacy and place in therapy. Cancer Manag Res 17:743–756 40196851 10.2147/CMAR.S441550 PMC11974566

[19] Malterud K, Siersma VD, Guassora AD (2016) Sample size in qualitative interview studies: guided by information power. Qual Health Res 26(13):1753–1760 26613970 10.1177/1049732315617444

[20] Huang LM et al (2024) A qualitative study on illness perception and coping behaviors among patients with chronic obstructive pulmonary disease: implications for intervention. Int J Chron Obstruct Pulmon Dis 19:2467–2479 39583958 10.2147/COPD.S473790 PMC11584336

[21] Breivik E, Ervik B, Kitzmüller G (2025) Preparing for home death in rural areas - the experience of family caregivers providing palliative cancer care. Int J Circumpolar Health 84(1):2507443. 10.1080/22423982.2025.2507443 40388215 PMC12090319

[22] Sands JM et al (2025) Practical management of adverse events in patients receiving tarlatamab, a delta-like ligand 3–targeted bispecific T-cell engager immunotherapy, for previously treated small cell lung cancer. Cancer 131(3):e35738 39876075 10.1002/cncr.35738 PMC11775405

[23] Hong C et al (2024) MASCC/ISOO clinical practice statement: management of salivary gland hypofunction and xerostomia in cancer patients. Support Care Cancer 32(8):548 39048728 10.1007/s00520-024-08688-9 PMC11550240

[24] Pan D et al (2025) Emerging GPRC5D-targeted therapies for multiple myeloma: a comprehensive review. Expert Opin Investig Drugs 34(5):379–389 40425184 10.1080/13543784.2025.2511179

[25] Rodriguez-Otero P et al (2024) GPRC5D as a novel target for the treatment of multiple myeloma: a narrative review. Blood Cancer J 14(1):24 38307865 10.1038/s41408-023-00966-9 PMC10837198

[26] Basch E et al (2016) Symptom monitoring with patient-reported outcomes during routine cancer treatment: a randomized controlled trial. J Clin Oncol 34(6):557–565 26644527 10.1200/JCO.2015.63.0830 PMC4872028

[27] Basch E et al (2025) Symptom monitoring with electronic patient-reported outcomes during cancer treatment: final results of the PRO-TECT cluster-randomized trial. Nat Med 31(4):1225–1232 39920394 10.1038/s41591-025-03507-y PMC12184200

[28] Gunn L et al (2021) Taste dysfunction following radiotherapy to the head and neck: a systematic review. Radiother Oncol 157:130–140 33545253 10.1016/j.radonc.2021.01.021

[29] van der Werf A et al (2018) Taste alterations during treatment with protein kinase inhibitors: a pilot study. J Pain Symptom Manage 56(4):e1–e4 30025935 10.1016/j.jpainsymman.2018.06.016

[30] Scordo M et al (2022) A prospective study of dysgeusia and related symptoms in patients with multiple myeloma after autologous hematopoietic cell transplantation. Cancer 128(21):3850–3859 36041227 10.1002/cncr.34444 PMC10010839

[31] Al Hadidi S et al (2025) Talquetamab in relapsed refractory multiple myeloma: multi-institutional real-world study. Blood Cancer J 15(1):196 10.1038/s41408-025-01386-7 41203605 PMC12594773

[32] Frenking JH et al (2025) A German multicenter real-world analysis of talquetamab in 138 patients with relapsed/refractory multiple myeloma. Hemasphere 9(4):e70114 40248128 10.1002/hem3.70114 PMC12005056

